# Adherence to daily electronic symptom screening in pediatric cancer: associations with time, location and pain

**DOI:** 10.1007/s11136-025-04002-0

**Published:** 2025-06-10

**Authors:** Jonas Dreher, Andreas Meryk, Johannes M. Giesinger, Verena Schneeberger-Carta, David Riedl, Alexandra Haid, Gabriele Kropshofer, Benjamin Hetzer, Gerhard Rumpold, Bernhard Holzner, Roman Crazzolara, Jens Lehmann

**Affiliations:** 1 University Hospital of Psychiatry II, Medical University of Innsbruck, Anichstraße 35, Innsbruck, AT-6020 Austria; 2 Department of Pediatrics, Medical University of Innsbruck, Anichstraße 35, Innsbruck, AT-6020 Austria; 3 University Hospital of Psychiatry I, Medical University of Innsbruck, Innsbruck, Austria

**Keywords:** Pediatric cancer, Patient-reported outcome measures (PROMs), Electronic health records, Symptom monitoring, Treatment adherence and compliance

## Abstract

**Purpose:**

Regularly collected patient-reported outcome measures (PROMs) can facilitate early symptom detection and improve health outcomes. This explorative analysis aimed to investigate PROM adherence and factors associated with daily PROM completion among pediatric cancer patients.

**Methods:**

We analyzed data from a prospective study at the Medical University of Innsbruck in which pediatric patients with cancer treated with chemotherapy completed daily PROMs via a web-based portal (ePROtect). We analyzed the PROM adherence during their first year of treatment descriptively and using a linear mixed model to evaluate factors associated with PROM adherence.

**Results:**

Fifty patients (42% female) with a median age of 10.7 years (IQR 7.1–15.4) were included in this analysis (analysis period 05/2020 to 05/2023). The mean adherence was 48.7% (SD 27.2), with the highest adherence during the first 30 days (77.1%). Significant predictive factors for lower adherence included time in the program (logarithmic; β = -0.093, *p* < 0.001) and admission to the intensive care unit (β = -0.224, *p* < 0.001). In contrast, inpatient stays (β = 0.035, *p* = 0.014) and self-reported pain (β = 0.087, *p* = 0.002) were significant predictors for higher PROM adherence. Occurrences of adverse events were not significantly associated with adherence.

**Discussion:**

Our findings suggest that continuous daily symptom monitoring is feasible over extended periods. PROM adherence stabilized over time despite an initial drop, with higher participation observed during inpatient stays and in weeks with self-reported pain. Future research should explore and evaluate strategies to improve adherence.

**Supplementary Information:**

The online version contains supplementary material available at 10.1007/s11136-025-04002-0.

## Plain language summary

Pediatric cancer patients undergoing treatment face numerous side effects that require continuous monitoring, and traditional methods can be inadequate. This study investigates how often young patients complete daily electronic symptom questionnaires and identifies factors that are influencing PROM adherence. We found that about half of the scheduled daily symptom reports were completed. The duration in the symptom monitoring program and admission to intensive care were linked to lower adherence, while inpatient stays and self-reported pain were associated with higher rates. These results suggest that continuous symptom monitoring is feasible for pediatric cancer patients but highlight the need for strategies to maintain high PROM adherence over time. Understanding these patterns and factors can help improve symptom tracking and care for these patients.

**Supplementary Information:**

The online version contains supplementary material available at 10.1007/s11136-025-04002-0.

## Background

Caring for pediatric cancer demands a comprehensive understanding of their health status throughout the course of treatment. Unlike standard evaluations by physicians and nurses, patient-reported outcomes (PROs) directly capture the subjective experiences of cancer patients in a structured manner [1]. Commonly, PROs can be easily assessed remotely using electronic systems, thus allowing patients’ health status to be monitored even if they are not in the hospital [2]. In adult patients with cancer, regular collection of PROMs during treatment has shown to improve patients’ overall survival, health-related quality of life (HRQOL), symptom control, and reduce hospitalizations and emergency room visits [3–6]. So far, such PRO monitoring systems are mainly used in clinical oncology studies with adult patients and less frequently implemented in routine clinical practice and pediatric patient populations [7]. In summary, there is growing evidence for electronic and mobile health interventions in pediatric patients with cancer, but no widespread implementation [8]. This demonstrates that the potential benefits of PRO monitoring systems are not yet fully realized for children with cancer, who are ideal candidates for PRO monitoring as they frequently undergo long and intensive therapies [9]. The accompanying side effects enable many possibilities where monitoring can be useful. Accordingly, PRO monitoring is recommended in acute care and survivorship for pediatric patients with cancer [10] and first projects are emerging piloting the integration of PROs into clinical care [11, 12]. Especially during outpatient treatment, regular electronic PRO monitoring with a self-owned device (i.e. smartphone, tablet, laptop) can be useful to monitor key symptoms and patients’ health from home [3, 13, 14]. Recently, two large-scale randomized controlled trials (RCTs) among pediatric patients with cancer demonstrated the potential benefits of electronic PRO monitoring for reducing patient symptom burden [15, 16].

One key consideration is how often PROMs should be assessed and which factors are associated with regularly completing PROMs. Symptom monitoring programs in adults [17, 18] often use weekly intervals to administer questionnaires to patients. Generally, these intervals are a compromise between achieving the best possible coverage of symptom trajectories (i.e., more frequent assessments) and minimizing patient burden (i.e., less frequent assessments) [19, 20]. However, there is evidence that when the aim is to capture patient-reported adverse events, relying on more frequent assessments is superior; a recent retrospective analysis of clinical trial data showed how using weekly assessments, only 50% of detectable severe symptoms could be detected, compared to 85% when assessments were conducted every other day [21]. Such analyses make a compelling case for more frequent assessments, especially for patient populations that are more confident in using electronic systems and the internet, such as children and young adolescents. The aforementioned RCTs among pediatric patients with cancer successfully used a daily symptom-monitoring (even if only for a short period of 5 consecutive days) and a thrice-weekly assessment schedule [15, 16]. While these trials offer promising evidence for both the feasibility and efficacy of conducting regular PRO monitoring, it remains important to evaluate which patient-level factors are associated with patients’ adherence to regular PROM completion, henceforth referred to as “PROM adherence”. Potentially PROM adherence may develop and change over time, depending on factors beyond the regularity of the assessment schedule (like technological or socioeconomic challenges). More research is needed to find the most appropriate PROM monitoring schedules for different settings.

At the Department of Pediatrics at the Medical University of Innsbruck, we have previously developed and evaluated a daily web- and mobile-based PROM monitoring program (ePROtect) [22, 23]. Prior results have already shown promising results for the early detection of adverse events [24]. Although the overall PROM adherence in our previous studies was high, when considering assessments were scheduled daily we also noted a decline over time in PROM adherence to the monitoring after 90 days [23]. This is a problem also observed in other eHealth interventions; adherence tends to start out high but drops with time due to attrition and also shows high variance among users [25–27]. Relevant factors influencing adherence in adults, and even less so in specific populations like children with cancer are however, not sufficiently researched [28]. In this study, we aim to (1) offer a more precise look into the trajectories of childrens’ PROM adherence to daily PROM completion over time and (2) investigate which factors influence adherence (e.g., age, sex, in- vs. outpatient treatment, presence of adverse events, time in the program).

## Methods

### Study design

Building on our previous study (see Meryk et al. [23]), we conducted an exploratory analysis with additional data that was collected since our original publication. The use of these data is covered by an approval of the ethics committee of the Medical University of Innsbruck (Austria) (EC No.: 1055/2020). The study was conducted in accordance with the declaration of Helsinki and all parents and patients provided written informed consent. For this study we followed the Strengthening the Reporting of Observational Studies in Epidemiology (STROBE) cohort checklist that can be found in Supplementary Material 1.

### Setting and participants

We analyzed data collected between 08.05.2020 (earliest start date) and 15.05.2023 (date of data extraction) at the Department of Pediatrics at the Medical University of Innsbruck. Data for the study were prospectively collected as part of a study accompanying routine clinical care. The ePRO system (ePROtect) was first implemented in May 2020. In brief, the program encompassed daily electronic collection of PROMs to monitor key symptoms and using the data in daily clinical care. The system and use of ePROtect are described in the section ‘daily PROM assessments’ and in more detail in Meryk et al. [23, 24].

All patients and their families with sufficient German-language proficiency, no cognitive disability and no visual impairments that precluded use of web application were approached for participation in the ePROtect program at the Department of Pediatrics at the Medical University of Innsbruck. For this analysis, we included patients whose parents had provided written informed consent and who received chemotherapy treatment and participated in the ePROtect program for at least the first 30 days after inclusion. This minimum time frame was chosen to best represent the clinical use of the ePROtect system, which involves long-term and continuous use (i.e., focus on patients who had been in the program for a long time). We did not include patients for whom only proxy reports were completed (e.g., younger than 5 years of age). For this analysis, we considered patients’ data for up to one year per patient (some patients remained in the program longer) or until:

- The patient’s chemotherapy had ended (in most cases, removal of the central venous catheter), which was the end of their daily PROM schedule and they transitioned to follow-up care with prolonged PROM schedules, or.
- Until the patient transitioned to receive treatment at a different hospital, or.
- Until death if the patient had deceased within the analysis period.

### Daily PRO assessments and clinical use

As part of the ePROtect program, patients were instructed to complete a short questionnaire every day on the ePROtect platform which was available as a website for computers and mobile devices [23]. ePROtect was developed based on the Computer-based Health Evaluation System (CHES) [29], a software that has been used at the Innsbruck Medical University in similar adult oncological clinical applications of PROM monitoring [29–32]. Patients were instructed to complete the questionnaire regardless of whether they were in inpatient or outpatient care at the time of the scheduled assessment. The questionnaire is described in more detail in the original paper [23] and assesses a total of 6 questions on the domains pain (1 item), nausea and appetite loss (2 items), sleep disturbance (1 item), and physical functioning (2 items).

Symptom data from the PROMs were reviewed daily by the healthcare team: If patients were at the hospital, their data were discussed at the ward rounds. The same applied to patients in outpatient care. The attending physician reviewed the PROMs and, if scores had deteriorated, could initiate appropriate clinical interventions (see, e.g [23, 24]).,.

### Data

Patients’ sociodemographic and clinical data were collected at the day of inclusion in ePROtect. For every day in the ePROtect program, we assessed whether patients had completed or not completed the scheduled PROMs. Moreover, we extracted patients’ treatment location on that day from the hospital records (i.e., the location of care: outpatient, inpatient, unplanned hospitalization, ambulatory care, pediatric intensive care unit). Following our standard of care, for every day that patients received care at the hospital, either as outpatients or inpatients, adverse events were assessed via standardized physical examinations and, as applicable, laboratory checks and documented based on the Common Terminology Criteria for Adverse Events (CTCAE) version 5 [33]. We assessed the most common adverse events during therapy and categorized them into events for which a grade ≥ 3 is considered clinically acute (febrile neutropenia, mucositis, pneumonia) as well as events that are more slowly progressing and are routinely monitored and diagnosed in a laboratory (hepatopathy and anemia). Finally, we also included patients’ PROM scores in the analysis by measuring whether patients reported symptoms met pre-defined thresholds indicating severe or very severe symptom burden [23] on a specific day.

### Statistical analysis

Descriptive statistics are reported for the sample characteristics (e.g., demographic information, clinical information). The adherence to PROM completion was calculated as the number of completed PROM assessments divided by the number of expected PROM assessments for each patient, which was equal to the number of days in the program. As patients were in the program for a varying amount of days, we first calculated the total daily PROM adherence for each patient individually and then averaged it across patients. As a sensitivity analysis, we calculated the weekly PROM adherence for each patient in which we considered weeks where at least 3 or more assessments were completed as a complete week. This weekly PROM adherence better conveys in which weeks at least some PRO coverage was available to the clinical team.

In the analysis to identify factors influencing patients’ PROM adherence, we considered weekly intervals of patients’ PROM adherence. This time frame was chosen as it most accurately conforms to the nature of our data. Many patients are at home for prolonged periods of time during treatment but commonly have at least one hospital appointment each week where blood samples are collected and CTCAE data are recorded. We used linear mixed models to identify factors linked to patients’ weekly PROM adherence. Table 1 gives an overview of the covariates that were considered and how they were defined. As a first step, we ran univariate models with each covariate as a fixed effect and the patient as the random effect with the patient’s weekly PROM adherence as the dependent variable. We tested the model fit for ‘number of weeks’ as both a linear factor as well as a logarithmic factor, as the data indicated logarithmic decline in PROM adherence over time, rather than a linear decrease. Significant predictors (p < 0.05) in these models were then added to a multivariable model with the significant covariates as fixed effects, the patient as random effect, and the patient’s weekly PROM adherence as the dependent variable. In addition to this strategy, we also created a full model with all covariates included as a sensitivity analysis. Analyses were run in RStudio, using the package “lme4” to perform the mixed linear regressions [34], and “MuMin” to calculate the Marginal R² and Conditional R² [35].

**Table 1:** Covariates considered in regression models to identify factors linked to patients’ weekly PROM adherence

Covariate	Description	Data type
Time in the program (log)	Number of weeks the patient had been in the program at that point in time	Integer (e.g., 12 weeks in the program, fitted as a logarithmic variable)
Age	Age of the patient on the day of inclusion in the ePROtect program	Double (e.g., 8.5 years)
Sex	Biological sex assigned at birth	Binary (Male/Female)
Caregiver highest education	Highest education achieved of any caregiver	Integer (Higher number equals higher education)
Urban residency	Defined as living in a municipality with more than 10,000 inhabitants.	Binary (Urban/Rural)
Tyrol residency	Defined as either living inside the state of Tyrol or outside (both within Austria and abroad)	Binary (Tyrol/not Tyrol)
Single parent	Defined as living in a household without a cohabiting partner or spouse.	Binary (Single parent/not single parent)
Number of siblings	Number of siblings of the patient	Integer (i.e. 2 other siblings; not counting the patient themselves)
Location(s) of care during the week (multiple could apply)		
Outpatient	If the patient spent 4 or more days at home that week	Binary (true/false)
Inpatient	If the patient spent 2 or more days as an inpatient that week	Binary (true/false)
Unplanned hospitalization	If the patient was admitted to the hospital for 1 or more days due to complications that week	Binary (true/false)
PICU	If the patient spent 1 or more days in the PICU that week	Binary (true/false)
Ambulatory care	If the patient visited the hospital 1 or more days for routine medical checkups **and** treatment that week	Binary (true/false)
CTCAE events (graded according to CTCAE v5, in a given week, only one could apply)		
Max Clinically acute CTCAE grade < = 2	If the patient had at least one grade 2 CTCAE event and below (CTCAEs assessed: febrile neutropenia, mucositis, pneumonia) and did not have any higher grade CTCAEs	Binary (true/false)
Max Clinically acute CTCAE grade > = 3	If the patient had at least one grade 3 CTCAE event and above (CTCAEs assessed: febrile neutropenia, mucositis, pneumonia and did not have any higher grade CTCAEs	Binary (true/false)
Max Laboratory diagnosed CTCAE grade < = 2	If the patient had at least one grade 2 CTCAE event and below (CTCAEs assessed: anemia, hepatopathy) and did not have any higher grade CTCAEs	Binary (true/false)
Max Laboratory diagnosed CTCAE grade > = 3	If the patient had at least one grade 3 CTCAE event and above(CTCAEs assessed: anemia, hepatopathy) and did not have any higher grade CTCAEs	Binary (true/false)
PROM scores		
PRO Pain	If the patient reported severe (score of 26–50 on a 0-100 scale) or very severe pain (score of 0–25) 1 or more days that week	Binary (true/false)
PRO Nausea	If the patient reported severe (score of 26–50 on a 0-100 scale) or very severe nausea (score of 0–25) 1 or more days that week	Binary (true/false)
PRO Sleep	If the patient reported severe (score of 26–50 on a 0-100 scale) or very severe sleep disturbances (score of 0–25) 1 or more days that week	Binary (true/false)
PRO Physical Function	If the patient reported (score of 26–50 on a 0-100 scale) or very severe problems with physical functioning(score of 0–25) 1 or more days that week	Binary (true/false)

Note. PICU: Pediatric intensive care unit; CTCAE: Common Terminology Criteria for Adverse Events; PRO: Patient-reported outcome, PROM: Patient-reported outcome measure

## Results

### Patient characteristics

**Fig. 1:**
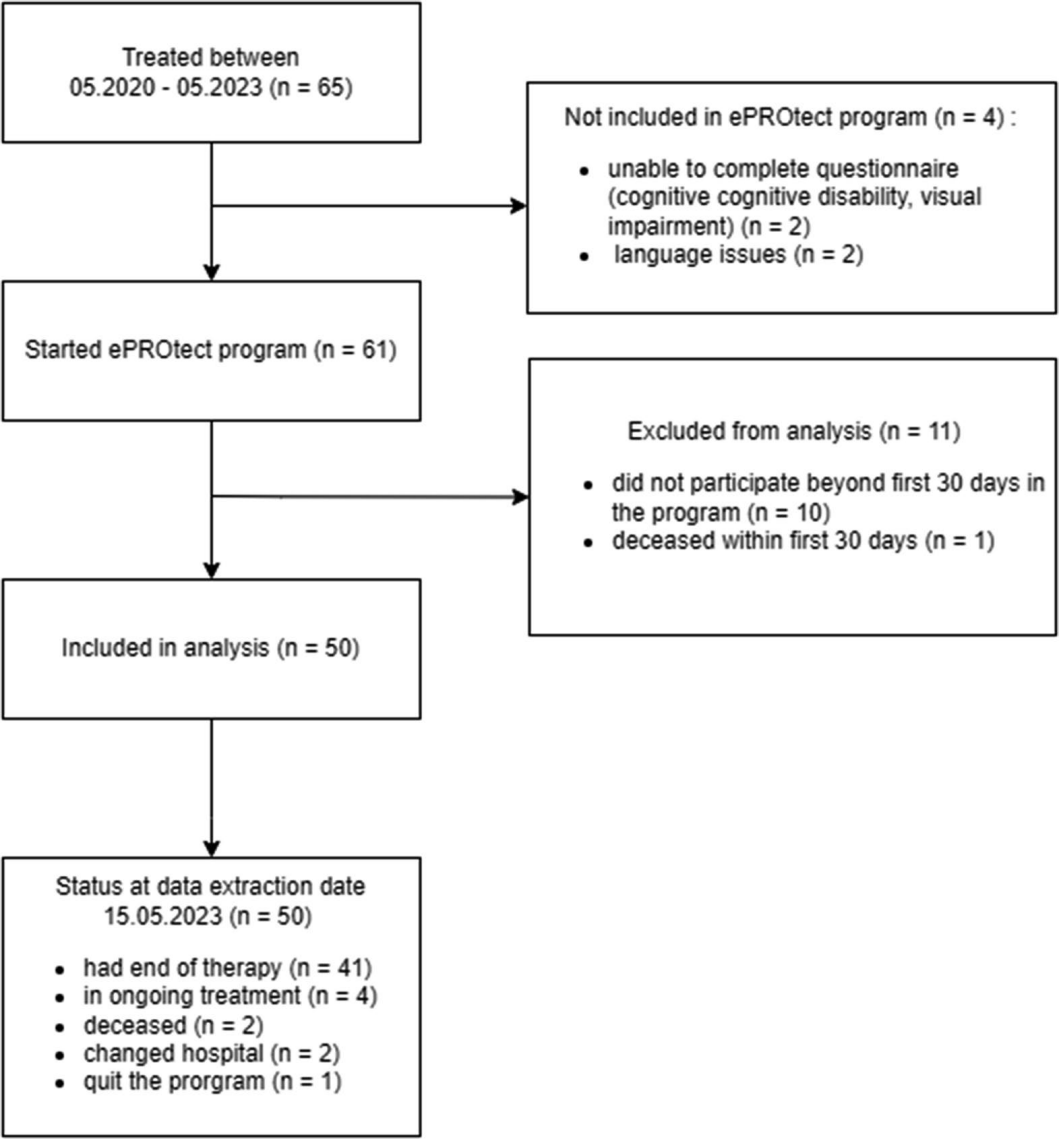
Analysis flowchart

During the period considered in the analysis, 61 out of 65 (94%) patients treated with chemotherapy were approached to participate in the ePROtect program. Reasons for not approaching patients (*n* = 4) were language barriers or cognitive impairments. Figure 1 shows the complete inclusion and study flowchart. Of 61 patients who started the program, 50 patients were considered for the final analysis. At the time of data extraction, 41 (82.0%) patients had completed their treatment, 4 (8.0%) were in ongoing treatment, 2 (4.0%) had passed away, another 2 (4.0%) had transitioned to care in another hospital and ended the ePROtect program and 1 (2.0%) quit the program.

Patients had a median age of 10.7 years (IQR 7.1–15.4). Most patients (*n* = 29, 58%) were male and 21 (42%) were female. The most prevalent cancer diagnoses were leukemia (acute lymphoblastic leukemia *n* = 15, 30%; acute myeloid leukemia *n* = 1, 2%), followed by lymphoma (*n* = 12, 24%), germ cell tumors (*n* = 5, 10%), and other types of cancer (*n* = 5, 10%). Socioeconomically, the majority of caregivers obtained an education below high-school level (*n* = 35, 70%), while residing in urban areas (*n* = 35, 70%). Half of them (*n* = 25, 50%) resided within Tyrol and approximately a third are single-parent households (*n* = 15, 30%).

**Table 2:** Patient characteristics

	Sample (*n* = 50)	
**Age in years**		
Median, IQR	10.70	7.1–15.4
Min, Max	5.00	17.98
**Sex (N**,** %)**		
Male	29	58.0
Female	21	42.0
**Cancer type (N**,** %)**		
Leukemia	16	32.0
ALL	15	(30.0)
AML	1	(2.0)
Hodgkin lymphoma	8	16.0
GCT	5	10.0
CNS tumor	4	8.0
STS	4	8.0
NHL	4	8.0
Bone tumor	4	8.0
Other	5	10.0
**Caregiver highest education**		
Below High School level	35	70.0
High School Diploma	5	10.0
University Degree	10	20.0
**Urban residency**		
Urban (> 10.000 inhabitants)	35	70.0
Rural (< 10.000 inhabitants)	15	30.0
**Tyrol residency**		
Living in Tyrol	25	50.0
Living outside Tyrol	25	50.0
**Single Parent**		
Single Parent Household	15	30.0
Multiple Parent Household	35	70.0
**Number of siblings**		
0	10	20.0
1	25	50.0
2	9	18.0
3	6	12.0
**Time in study (average days per patient**,** SD)**		
Overall	221.4	97.5
At home	130.7	72.8
Inpatient	45.1	29.5
Ambulatory care	27.1	24.2
Unplanned Hospitalization	15.9	20.8
Picu	1.3	4.4

Note. ALL = acute lymphoblastic leukemia, AML = acute myeloid leukemia, GCT = Germ cell tumor, CNS = Central nervous system, STS = Soft-tissue sarcoma, NHL = Non-Hodgkin lymphoma, PICU = Pediatric intensive care unit

**Fig. 2:**
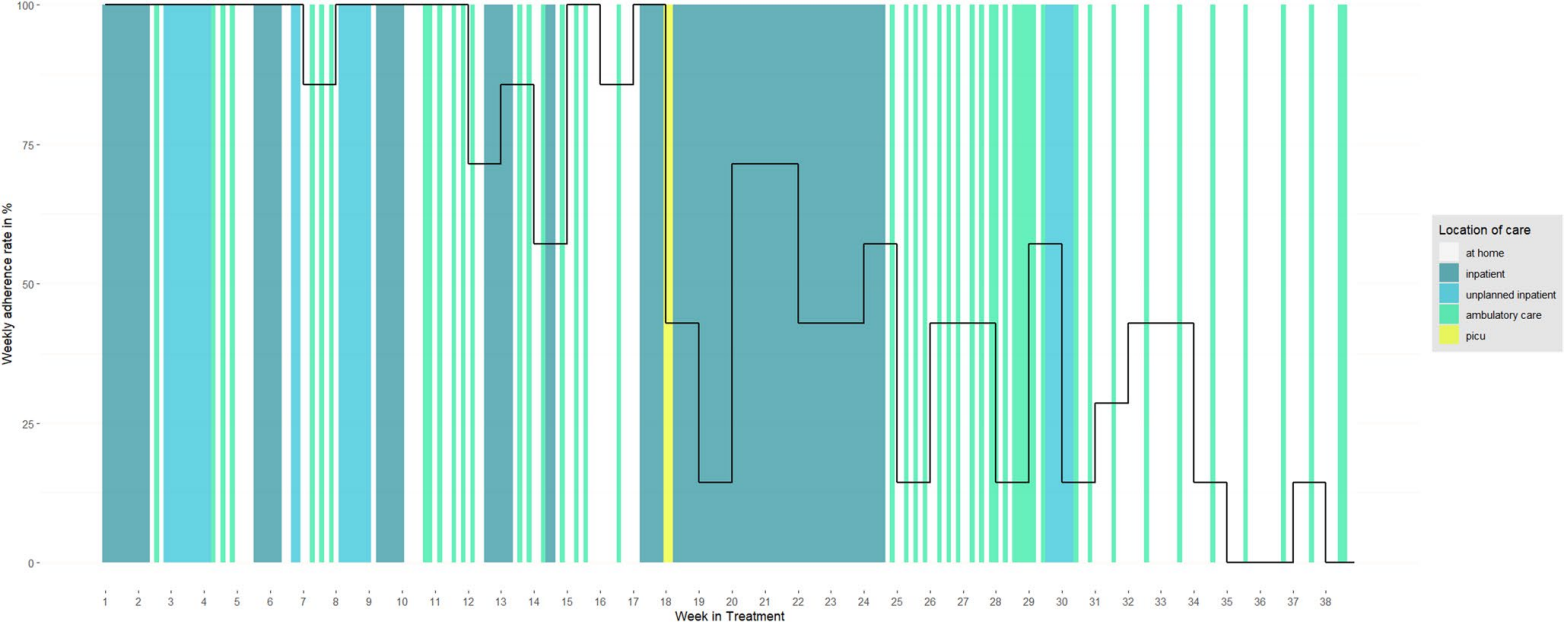
Weekly PROM adherence and daily location of care from a single patient over time. Note. The graphic follows the treatment of a male patient with t-cell acute lymphoblastic leukemia

Across all patients, a total of 12,154 days in the program were analyzed. On 7,031 of those days (58%), patients were at home and remotely monitored using PROMs. For the remaining 5,123 days (42%), patients were located in various medical care arrangements within a hospital (i.e. inpatient, ambulatory, etc.). Additional details on patient characteristics are provided in Table 2. Patients frequently transitioned between receiving ambulatory care, periods at home, and inpatient stays. To illustrate, Fig. 2 shows the weekly PROM adherence of a patient with acute lymphoblastic leukemia and changes in treatment location during the time in study; initial completion was high, but then slowly decreased after the first 30 days (corresponding to the therapy consolidation period) and during post-consolidation (after day 60).

Patients in our study were in the analysis period for an average of 221 days (SD = 98). On average, patients spent most days in an outpatient setting at home (mean of 131 days, SD 73), followed by inpatient stays (45 days, SD 30) and a smaller portion of days receiving ambulatory care (27 days, SD 24). Patients less frequently had days with unplanned hospitalizations (16 days, SD 21) or the pediatric intensive care unit (PICU, 1 day, SD 4).

During the study period, 86% (*n* = 43) of patients had at least one grade 3 CTCAE event of any type, and 30% (*n* = 15) had at least one grade 4 CTCAE event. More data on the distribution of CTCAE events is shown in Supplementary Material 2.

### Adherence to daily PROM completion

Patients’ mean daily PROM adherence was 48.7% with the highest average PROM adherence during the first 30 days (77.1%) and lower average PROM adherence in days 31–60 (56.4%) and days 61–365 (42.2%). When considering completion on a weekly basis and considering weeks where at least 3 or more assessments were completed (indicating at least some PROM coverage of that week from a clinical perspective), patients had a PROM adherence of 59.5%. Additional subgroup analysis by age groups show only minor differences between the groups. More details can be found in the *Supplementary Material 2.*

**Fig. 3:**
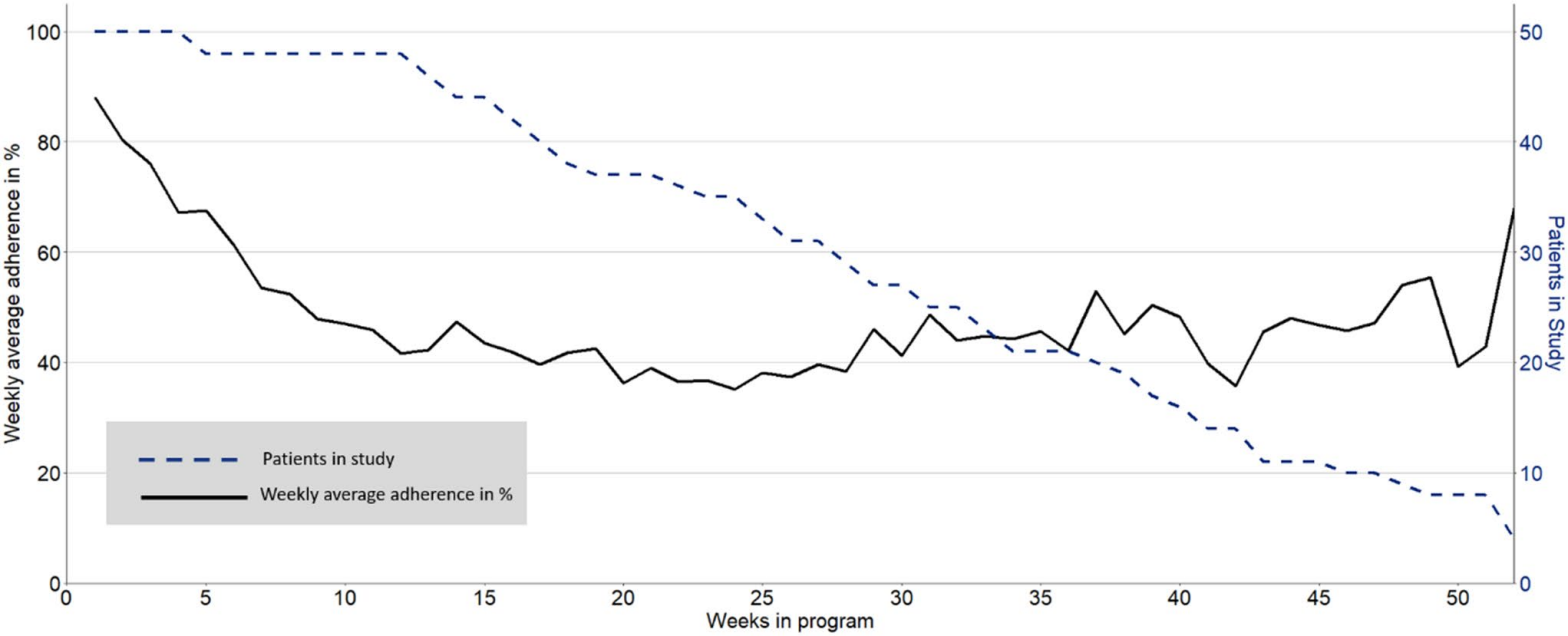
Weekly average PROM adherence over time of all patients

Patient PROM adherence varies by location of care, as detailed in Table 3. PROM adherence were found to differ between locations of care with the highest mean completion reported at the inpatient setting (mean adherence 58.8%), followed by unplanned hospitalization stays (53.7%). In comparison, patients had a mean PROM adherence of 45.2% on outpatient days. Figure 3 shows the mean weekly PROM adherence for each week and changes thereof while accounting for the number of patients. PROM adherence was highest at inclusion, gradually decreasing up until week 10 and then stabilizing around 50% after week 10.

**Table 3:** PROM adherence average across patients and by location of care

PROM Adherence (percent)	Mean	SD
Daily^a^	48.7	27.2
Weekly^b^	59.5	31.5
**Mean daily PROM adherence (at location of care)**		
Inpatient	58.8	26.1
Unplanned hospitalization	53.7	32.6
Ambulatory care	45.4	33.3
At home	45.2	31.5
PICU	31.7	40.0

Note. PICU = Pediatric intensive care unit; ^a^In this analysis, PROM adherence was considered on a daily basis per patient; ^b^In this sensitivity analysis, we considered PROM adherence on a weekly basis with weeks with 3 assessments or more as adherent

### Regression models to identify factors associated with weekly PROM completion

We modeled time in the program as a logarithmic factor based on better fit indices compared to a linear factor (data not shown) and visual inspection of the data which indicated a decline up until week 10 (Fig. 3). Table 4 shows the results from the univariate and multivariable models.

In the univariate models, the covariates *time in program* (β= -0.1500, *p* < 0.001), *outpatient* (β= -0.0844, *p* < 0.001) and *PICU* (β= -0.1408, *p* = 0.027) showed a statistically significant negative effect on weekly PROM adherence. The factors *Inpatient* (β = 0.1007, *p* < 0.001) and *PRO Pain* (β = 0.0773, *p* = 0.015) showed a statistically significant positive effect on PROM adherence.

Significant factors were combined in a multivariable model. The factors *time in program (*β*=* -0.0933, *p* < 0.001), *Inpatient (*β = 0.0345, *p* = 0.014), *picu* (β= -0.2239, *p* < 0.001), *PRO Pain* (β = 0.0873, *p* = 0.002) were statistically significant predictors of patients’ weekly completion. Considering that time was modeled as a logarithmic variable, this means that for every unit increase in the logarithm of *time in program*, the PROM adherence decreased by about 9.3%. In other words, after the two weeks PROM adherence decreased by 6.5%, after five weeks by 15.0%, after 26 weeks by 30.4%, and after 52 weeks by 36.9%. Additionally, in weeks where patients had inpatients stays or were admitted in the PICU, they had a 3.4% higher average PROM adherence or 22.4% lower, respectively.

The sensitivity analysis, which included all predictors in a full model, yielded results that were both consistent with and different from the multivariable model. *Time in program (*β*=* -0.0660, *p* < 0.001) and *picu* (β*=* -0.2466, *p* < 0.001) remained significant predictors with similar weights. However, *inpatient* (β *=* 0.1373, *p* = 0.105) and *PRO pain* (β *=* 0.0908, *p* = 0.134) were no longer statistically significant with an overall poorer model fit. The equation of all models and fit indices are reported in Supplementary Material 2.

All other covariates, including age, sex, all socioeconomic variables, some locations of care (unplanned hospitalization, ambulatory care), all CTCAE events, and some PRO measures (nausea, sleep, physical functioning) were not statistically significant in either the univariate and multivariate analyses.

**Table 4:** Univariable and multivariable regression models for patients’ weekly PROM adherence

Covariates	Univariate models			Multivariable model		
	β	[95% CI]	*p*-value	β	[95% CI]	*p*-value
Constant of multivariable model				**0.8158**	**[0.7459; 0.8858]**	**< 0.001**
Age	-0.0027	[-0.0212; 0.0157]	0.733			
Sex	-0.0887	[-0.2434; 0.0660]	0.266			
Caregiver highest education	0.0063	[-0.0900; 0.1025]	0.899			
Urban residency	-0.0237	[-0.1936; 0.1461]	0.785			
Tyrol residency	0.0901	[-0.0641; 0.2442]	0.258			
Single parent	-0.0190	[-0.1918; 0.1537]	0.830			
Number of siblings	-0.0301	[-0.1178; 0.0576]	0.504			
**Time in the program**	**-0.1500**	**[-0.1636; -0.1364]**	**< 0.001**	**-0.0933**	**[-0.1072; -0.0794]**	**< 0.001**
*Location of care*						
** At home**	**-0.0844**	**[-0.1147; -0.0541]**	**< 0.001**	-	-	n.s.
** Inpatient**	**0.1007**	**[0.0711; 0.1303]**	**< 0.001**	**0.0345**	**[0.0071; 0.0618]**	**0.014**
Unplanned hospitalization	0.0022	[-0.0450; 0.0450]	0.918			
** PICU**	**-0.1408**	**[-0.2652; -0.0164]**	**0.027**	**-0.2239**	**[-0.3317; -0.1161]**	**< 0.001**
Ambulatory care	-0.0180	[-0.0472; 0.0117]	0.238			
CTCAE Events						
Clinically acute CTCAE grade < = 2	-0.0471	[-0.1717; 0.0775]	0.460			
Clinically acute CTCAE grade > = 3	0.0192	[-0.0797; 0.6649]	0.704			
Laboratory diagnosed CTCAE grade < = 2	0.0091	[-0.2166; 0.2349]	0.937			
Laboratory diagnosed CTCAE grade > = 3	-0.0229	[-0.1168; 0.0711]	0.634			
*PROM scores*						
** PRO pain**	**0.0773**	**[0.0514; 0.1391]**	**0.015**	**0.0873**	**[0.0308; 0.1437]**	**0.002**
PRO nausea	0.0371	[-0.0337; 0.1079]	0.305			
PRO sleep	-0.0057	[-0.0914; 0.0801]	0.897			
PRO physical functioning	0.0434	[-0.0041; 0.0908]	0.074			

Note. Fit indices for the multivariable model: Akaike Information Criterion = -283.3; Bayesian Information Criterion = -202.9; Log-Likelihood = 126.1; Marginal R² = 0.11, Conditional R² = 0.58; PRO = Patient Reported Outcomes; PROM = Patient Reported Outcome Measure; CTCAE = Common Terminology Criteria for Adverse Events; β = unstandardized regression coefficient; Significant variables are highlighted in bold

## Discussion

In this explorative analysis, we evaluated pediatric patients’ adherence to daily scheduled PROMs while on treatment, called PROM adherence. The PROM adherence varied considerably between patients and over time, but patients generally showed high adherence to the PRO monitoring and completed questionnaires in most weeks even over long time periods (> 25 weeks). Relevant factors associated with patients’ PROM adherence were time in the program, the location of care, and self-reported pain, while patient socioeconomic factors, age, sex and the occurrence of adverse events were not associated with adherence.

### Measuring adherence and balancing frequent assessments with patient burden

Several of our findings merit in-depth discussion. The daily PROM adherence across all patients was 48%, meaning that patients on average completed half of all scheduled assessments. It is important to note that in more than half of the weeks in the program (59%), patients had at least three PROM assessments or more, where they still provided a lot of information on patients’ health status information. This number and decrease in completion over time is also similar to what was achieved in another trial testing weekly assessments over a longer time period [36]. While daily PROM completion is encouraged in our program, the reality shows this ideal scenario may not always be achievable. Moreover, research indicates that as long as a PROM assessment is provided every other day, more relevant symptoms can be detected compared to, for example, weekly assessments [21].

We provide different analyses of PROM adherence. A recent systematic review shows that most reporting of PROM adherence mainly refers to the percentage of completed questionnaires over the entire time-frame [26]. However, it is not clear whether this approach is the most suitable when evaluating long-term completion in routine care settings. Hence, we provide different evaluations of the PROM adherence. We report the most commonly used methodology in the overall completion as it gives an easy to compare and understandable result. In some cases decreasing PROM adherence does not necessarily have to be worrisome: For example, patients (and their caregivers) might stop completing PROMs if they are feeling well and do not see any benefits in providing daily updates if their situation does not change. Additionally, we considered the PROM adherence on a weekly basis as this was also deemed relevant duration for clinical use (i.e., providing regular health status information several times a week). This may be a useful measure of long-term PROM adherence as patients may complete assessments every other day, resulting in 50% PROM adherence when considered solely on a daily basis, but higher PROM adherence when considered on a weekly basis.

Striking a balance in the frequency of the monitoring will always be a challenge. From the healthcare team’s perspective, more frequent monitoring is often desirable, which can lead to high burden for patients [37]. From the patients’ perspective, burden from regular monitoring needs to be balanced with additional safety gained from the monitoring. In our case, our previous findings show that families of patients highly value the benefit of increased connectivity with the healthcare team, especially in the remote setting, which outweighs the burden of a regular (and very brief) PRO assessment [22].

### Factors associated with patients’ weekly PROM adherence

Unsurprisingly, time since the inclusion in the program was a relevant factor that was significantly negatively associated with patients’ PROM adherence in our multivariable model. Notably, time also acts as a surrogate marker for therapy intensity; measurements taken closer to the baseline reflect a higher intensity of therapy. At the same time, the duration and intensity of therapies also differed between patients depending on their diagnoses, which is something our model does not account for. Overall, a logarithmic function was the best fit for our data. Hence, decreases in PROM adherence over time were initially high but decreases were less pronounced in the long term. After 10 weeks, PROM adherence remained at around 50%, indicating that long-term completion is possible over longer periods of time.

Socioeconomic factors were not significantly associated with PROM adherence in our sample. While direct evidence on the role of socioeconomic characteristics in PROM adherence among pediatric oncology patients is limited, related research on treatment adherence suggests mixed findings [38–41]. For instance, in a large study of children with acute lymphoblastic leukemia, living in a single-parent household was associated with reduced adherence to oral maintenance therapy [40]. However, broader constructs like family cohesion may outweigh individual socioeconomic factors [41]. Our findings align with this perspective, suggesting that socioeconomic variables, while influential in some contexts, did not substantially affect PROM adherence in our study. This highlights the potential importance of interpersonal dynamics over structural factors in PROM adherence. Additionally, neither age nor sex were significant predictors of patients’ weekly PROM adherence. This stands in contrast to findings from studies with adults where both age and sex were significantly associated with PROM adherence (e.g [42]). While we cannot rule out that our sample was too small to find such effects, it is encouraging to see that PROM adherence was high even for our youngest patients (5–9 years) and that they could regularly self-report their health status. It seems sensible that these effects of age and sex are reduced or not present in a technology-savvy population like children whose parents also likely supported regular PROM completion. At the same time, it is also possible that not all children in this age group own a device themselves and that some rely on their parents.

Finally, both health status factors (self-reported pain) and the location of care were significant predictors of patients’ PROM adherence. During inpatient stays, which usually take place over longer time periods, higher PROM adherence was observed, although this effect was rather small. In our case, this is likely linked to the healthcare team actively supporting and reminding patients and families to complete PROMs. Moreover, during inpatient stays, clinicians regularly engaged with PROMs, which is known to increase patient adherence to completion [28, 43, 44]. By extension, this could mean that similar reminders, even ones provided by the application itself could further boost PROM adherence in other locations. These should be tailored to individual patient behaviors and treatment schedules and provide timely and context-sensitive prompts to complete PROMs [45]. Interestingly, CTCAE events were not significantly associated with PROM adherence. This stands in contrast with the sometimes proposed factor that patients might stop completing PROMs when they are too ill [28]. While patients admitted to the PICU had a significantly lower PROM adherence, this was not observed outside of the intensive care setting. Reassuringly we found that, in our sample, patients did not stop completing PROMs when they had moderate or severe CTCAEs. Instead, patients were significantly more likely to complete PROMs in weeks where they had higher self-reported pain. This may indicate that patients and their families used the PRO monitoring system to proactively self-report a deteriorated health status to the healthcare team.

### Strengths and limitations

Our study has several limitations. First, the retrospective and explorative analysis, as well as the single-center design limit the generalizability of our conclusions. Further limits to generalizability arise due to the presumed homogeneity in sociodemographic factors. We therefore encourage future research to conduct multicenter studies in different (cultural) contexts and settings.

Second, while we assessed some socioeconomic factors, we did not capture any higher-order family related variables such as social, cultural or emotional support, which may be linked to PROM adherence. In general, for younger patients, it is likely that completion is more proactively prompted by parents, which is something we did not control for. More details on the role of parents and interactions with childrens’ ratings are discussed in our other papers [22, 46].

The analysis could also be improved by adding interaction terms into account. We chose not to include them in this analysis due to the already large number of predictors and the potential risk of overfitting given our sample size but encourage future research that are not facing this problem to also explore the interaction between predictors.

The main strengths of this paper are the quality of the data with daily PROM assessment over a long time period, as well as detailed assessment of CTCAEs in the inpatient setting and continued assessment of the clinical location of care.

## Conclusion and outlook

In summary, our study shows that, in a routine clinical care setting and over longer time periods, high PROM adherence and good PRO health status coverage can be achieved. Likely, a contributing factor is the strong integration of ePROtect into clinical care achieved in our department. With daily symptom assessments that are actively reviewed by clinicians, the patients and their caregivers are highly motivated to consistently engage with the system.

Symptom monitoring programs using PROMs are increasingly recognized and implemented in pediatric populations [10, 37]. At the same time, they are still not a standard of care. One important factor to increase uptake also outside of a clinical trial setting is to understand why and when patients and caregivers engage with such systems. Our findings give insight into which factors are associated with patients’ PROM adherence and may be valuable in this context.

Additionally, while for parents, improved communication with the healthcare team and a feeling of safety are important factors, gamification has been proposed as a means to better engage children in completing PROMs [10, 37]. Since concluding this analysis, we have updated the ePROtect interface for children and introduced a native smartphone app which includes gamification elements. While gamification is potentially promising tool for engaging pediatric populations, further research is needed to evaluate its long-term effectiveness and address arising issues, such as the difficulty of creating a one-size-fits all approach. Different age groups are likely to respond to different gamification strategies that require tailored solutions. Efforts are ongoing to compare PROM adherence once more data are available.

## Electronic supplementary material

Below is the link to the electronic supplementary material.

Supplementary Material 1

Supplementary Material 2

## References

[1] U.S. Department of Health and Human Services FDA Center for Drug Evaluation and Research, U.S. Department of Health and Human Services FDA Center for Biologics Evaluation and Research, & U.S. Department of Health and Human Services FDA Center for Devices and Radiological Health. (2006). Guidance for industry: patient-reported outcome measures: Use in medical product development to support labeling claims: Draft guidance. *Health and Quality of Life Outcomes*, *4*(1), 79. 10.1186/1477-7525-4-79 17034633 10.1186/1477-7525-4-79 PMC1629006

[2] Warrington, L., Absolom, K., Conner, M., Kellar, I., Clayton, B., Ayres, M., & Velikova, G. (2019). Electronic systems for patients to report and manage side effects of Cancer treatment: Systematic review. *Journal of Medical Internet Research*, *21*(1), e10875. 10.2196/10875 30679145 10.2196/10875 PMC6365878

[3] Basch, E., Deal, A. M., Kris, M. G., Scher, H. I., Hudis, C. A., Sabbatini, P., & Schrag, D. (2016). Symptom monitoring with Patient-Reported outcomes during routine Cancer treatment: A randomized controlled trial. *Journal of Clinical Oncology*, *34*(6), 557–565. 10.1200/JCO.2015.63.0830 26644527 10.1200/JCO.2015.63.0830 PMC4872028

[4] Denis, F., Basch, E., Septans, A. L., Bennouna, J., Urban, T., Dueck, A. C., & Letellier, C. (2019). Two-Year survival comparing Web-Based symptom monitoring vs routine surveillance following treatment for lung Cancer. *Journal of the American Medical Association*, *321*(3), 306. 10.1001/jama.2018.18085 30667494 10.1001/jama.2018.18085 PMC6439676

[5] Velikova, G., Absolom, K., Warrington, L., Morris, C., Hudson, E., Carter, R., & Brown, J. (2020). Phase III randomized controlled trial of eRAPID (electronic patient self-Reporting of Adverse-events: Patient information and advice)—An eHealth intervention during chemotherapy. *Journal of Clinical Oncology*, *38*(15_suppl), 7002–7002. 10.1200/JCO.2020.38.15_suppl.7002

[6] Barbera, L., Sutradhar, R., Seow, H., Earle, C. C., Howell, D., Mittmann, N., & Thiruchelvam, D. (2020). Impact of standardized Edmonton symptom assessment system use on emergency department visits and hospitalization: Results of a Population-Based retrospective matched cohort analysis. *JCO Oncology Practice*, *16*(9), e958–e965. 10.1200/JOP.19.00660 32463762 10.1200/JOP.19.00660

[7] Anatchkova, M., Donelson, S. M., Skalicky, A. M., McHorney, C. A., Jagun, D., & Whiteley, J. (2018). Exploring the implementation of patient-reported outcome measures in cancer care: Need for more real-world evidence results in the peer reviewed literature. *Journal of Patient-Reported Outcomes*, *2*(1), 64. 10.1186/s41687-018-0091-0 30588562 10.1186/s41687-018-0091-0 PMC6306371

[8] Ramsey, W. A., Heidelberg, R. E., Gilbert, A. M., Heneghan, M. B., Badawy, S. M., & Alberts, N. M. (2020). eHealth and mHealth interventions in pediatric cancer: A systematic review of interventions across the cancer continuum. *Psycho-Oncology*, *29*(1), 17–37. 10.1002/pon.5280 31692183 10.1002/pon.5280

[9] Butler, E., Ludwig, K., Pacenta, H. L., Klesse, L. J., Watt, T. C., & Laetsch, T. W. (2021). Recent progress in the treatment of cancer in children. *CA: A Cancer Journal for Clinicians*, *71*(4), 315–332. 10.3322/caac.21665 33793968 10.3322/caac.21665

[10] Horan, M. R., Sim, J., Krull, K. R., Ness, K. K., Yasui, Y., Robison, L. L., & Huang, I. C. (2023). Ten considerations for integrating Patient-Reported outcomes into clinical care for childhood Cancer survivors. *Cancers*, *15*(4), 1024. 10.3390/cancers15041024 36831370 10.3390/cancers15041024 PMC9954048

[11] Calligan, M., Chakkalackal, L., Dadzie, G., Tardif-Theriault, C., Cook, S., Vettese, E., & Sung, L. (2023). Feasibility of three times weekly symptom screening in pediatric cancer patients. *Bmc Cancer*, *23*(1), 4. 10.1186/s12885-022-10400-1 36597030 10.1186/s12885-022-10400-1 PMC9809057

[12] Van Muilekom, M. M., Teela, L., Van Oers, H. A., Van Goudoever, J. B., Grootenhuis, M. A., & Haverman, L. (2022). Patients’ and parents’ perspective on the implementation of patient reported outcome measures in pediatric clinical practice using the KLIK PROM portal. *Quality of Life Research*, *31*(1), 241–254. 10.1007/s11136-021-02950-x 34324137 10.1007/s11136-021-02950-x PMC8800898

[13] Richards, H. S., Blazeby, J. M., Portal, A., Harding, R., Reed, T., Lander, T., & Avery, K. N. L. (2020). A real-time electronic symptom monitoring system for patients after discharge following surgery: A pilot study in cancer-related surgery. *Bmc Cancer*, *20*(1), 543. 10.1186/s12885-020-07027-5 32522163 10.1186/s12885-020-07027-5 PMC7285449

[14] Daly, B., Nicholas, K., Flynn, J., Silva, N., Panageas, K., Mao, J. J., & Perchick, W. (2022). Analysis of a remote monitoring program for symptoms among adults with Cancer receiving antineoplastic therapy. *JAMA Network Open*, *5*(3), e221078. 10.1001/jamanetworkopen.2022.1078 35244701 10.1001/jamanetworkopen.2022.1078 PMC8897754

[15] Dupuis, L. L., Vettese, E., Grimes, A. C., Beauchemin, M. P., Klesges, L. M., Baggott,C.,… Sung, L. (2024). Symptom Screening Linked to Care Pathways for Pediatric Patients With Cancer: A Randomized Clinical Trial. *JAMA*, *332*(23), 1981. 10.1001/jama.2024.19585. 10.1001/jama.2024.19585 PMC11561721 39535768

[16] Dupuis, L. L., Johnston, D. L., Dix, D., McKillop, S., Cook, S., Crellin-Parsons,N.,… Sung, L. (2025). Symptom Screening for Hospitalized Pediatric Patients With Cancer: A Randomized Clinical Trial. *JAMA Pediatrics*, *179*(1), 11. 10.1001/jamapediatrics.2024.4727. 10.1001/jamapediatrics.2024.4727 PMC11561725 39535812

[17] Lai-Kwon, J., Cohen, J. E., Lisy, K., Rutherford, C., Girgis, A., Basch, E., & Jefford, M. (2023). The feasibility, acceptability, and effectiveness of electronic Patient-Reported outcome symptom monitoring for immune checkpoint inhibitor toxicities: A systematic review. *JCO Clinical Cancer Informatics*, (7), e2200185. 10.1200/CCI.22.00185 10.1200/CCI.22.00185 37220322

[18] Consolo, L., Castellini, G., Cilluffo, S., Basile, I., & Lusignani, M. (2022). Electronic patient-reported outcomes (e-PROMs) in palliative cancer care: A scoping review. *Journal of Patient-Reported Outcomes*, *6*(1), 102. 10.1186/s41687-022-00509-z 36138279 10.1186/s41687-022-00509-z PMC9500127

[19] King-Kallimanis, B. L., Bhatnagar, V., Horodniceanu, E. G., Chen, T. Y., & Kluetz, P. G. (2022). Timing is everything: The importance of patient-reported outcome assessment frequency when characterizing symptomatic adverse events. *Clinical Trials*, 174077452210939. 10.1177/17407745221093935 10.1177/17407745221093935 35575012

[20] Basch, E., Thanarajasingam, G., & Dueck, A. C. (2022). Methodological standards for using the patient-reported outcomes version of the common terminology criteria for adverse events (PRO-CTCAE) in cancer clinical trials. *Clinical Trials*, *19*(3), 274–276. 10.1177/17407745221093922 35575014 10.1177/17407745221093922

[21] Innominato, P. F., Komarzynski, S., Dallmann, R., Wreglesworth, N. I., Bouchahda,M., Karaboué, A.,… Lévi, F. A. (2021). Impact of assessment frequency of patient-reported outcomes: an observational study using an eHealth platform in cancer patients. *Supportive Care in Cancer*, *29*(11), 6167–6170. 10.1007/s00520-021-06262-1. 10.1007/s00520-021-06262-1 33963910

[22] Meryk, A., Kropshofer, G., Hetzer, B., Riedl, D., Lehmann, J., Rumpold, G., & Crazzolara, R. (2021). Implementation of daily patient-reported outcome measurements to support children with cancer. *Pediatric Blood & Cancer*, *68*(11). 10.1002/pbc.29279 10.1002/pbc.29279 34383360

[23] Meryk, A., Kropshofer, G., Hetzer, B., Riedl, D., Lehmann, J., Rumpold, G., & Crazzolara, R. (2022). Use of daily Patient-Reported outcome measurements in pediatric Cancer care. *JAMA Network Open*, *5*(7), e2223701. 10.1001/jamanetworkopen.2022.23701 35881395 10.1001/jamanetworkopen.2022.23701 PMC9327576

[24] Meryk, A., Kropshofer, G., Hetzer, B., Riedl, D., Lehmann, J., Rumpold, G., & Crazzolara, R. (2022). Bridging the gap in outpatient care: Can a daily patient-reported outcome measure help? *Cancer Reports*, *5*(1). 10.1002/cnr2.1421 10.1002/cnr2.1421 PMC8789604 34245127

[25] Colls, J., Lee, Y. C., Xu, C., Corrigan, C., Lu, F., Marquez-Grap, G.,… Solomon,D. H. (2021). Patient adherence with a smartphone app for patient-reported outcomes in rheumatoid arthritis. *Rheumatology*, *60*(1), 108–112. 10.1093/rheumatology/keaa202. 10.1093/rheumatology/keaa202 32572490

[26] Wiegel, J., Seppen, B., van der Leeden, M., van der Esch, M., de Vries, R., & Bos, W. (2021). Adherence to telemonitoring by electronic Patient-Reported outcome measures in patients with chronic diseases: A systematic review. *International Journal of Environmental Research and Public Health*, *18*(19), 10161. 10.3390/ijerph181910161 34639463 10.3390/ijerph181910161 PMC8508527

[27] Eysenbach, G. (2005). The law of attrition. *Journal of Medical Internet Research*, *7*(1), e11. 10.2196/jmir.7.1.e11 15829473 10.2196/jmir.7.1.e11 PMC1550631

[28] Unni, E., Coles, T., Lavallee, D. C., Freel, J., Roberts, N., & Absolom, K. (2023). Patient adherence to patient-reported outcome measure (PROM) completion in clinical care: Current Understanding and future recommendations. *Quality of Life Research*. 10.1007/s11136-023-03505-y 37695476 10.1007/s11136-023-03505-y PMC10784330

[29] Holzner, B., Giesinger, J. M., Pinggera, J., Zugal, S., Schöpf, F., Oberguggenberger,A. S.,… Rumpold, G. (2012). The Computer-based Health Evaluation Software (CHES):a software for electronic patient-reported outcome monitoring. *BMC Medical Informatics and Decision Making*, *12*(1), 126. 10.1186/1472-6947-12-126. 10.1186/1472-6947-12-126 PMC3529695 23140270

[30] Wintner, L. M., Giesinger, J. M., Zabernigg, A., Rumpold, G., Sztankay, M., Oberguggenberger,A. S.,… Holzner, B. (2015). Evaluation of electronic patient-reported outcome assessment with cancer patients in the hospital and at home. *BMC Medical Informatics and Decision Making*, *15*(1), 110. 10.1186/s12911-015-0230-y. 10.1186/s12911-015-0230-y PMC4690412 26699708

[31] Lehmann, J., Wintner, L. M., Sztankay, M., Willenbacher, W., Weger, R., Weyrer, W.,… Holzner, B. (2020). Patient-reported outcomes and psycho-oncological screening in hematology: a practical example of routine electronic monitoring. *memo - Magazine of European Medical Oncology*, *13*(3), 285–293. 10.1007/s12254-020-00628-7.

[32] Lehmann, J., Buhl, P., Giesinger, J. M., Wintner, L. M., Sztankay, M., Neppl, L.,… Holzner, B. (2021). Using the Computer-based Health Evaluation System (CHES) to Support Self-management of Symptoms and Functional Health: Evaluation of Hematological Patient Use of a Web-Based Patient Portal. *Journal of Medical Internet Research*, *23*(6), e26022. 10.2196/26022. 10.2196/26022 PMC8262597 34100765

[33] Common Terminology Criteria for Adverse Events (CTCAE) Version 5.0 (2017).

[34] Bates, D., Mächler, M., Bolker, B., & Walker, S. (2015). Fitting linear Mixed-Effects models using lme4. *Journal of Statistical Software*, *67*(1). 10.18637/jss.v067.i01

[35] Bartoń, K. (2010, May 28). MuMIn: Multi-Model Inference. 10.32614/CRAN.package.MuMIn

[36] Dussel, V., Orellana, L., Soto, N., Chen, K., Ullrich, C., Kang, T. I.,… Wolfe,J. (2015). Feasibility of Conducting a Palliative Care Randomized Controlled Trial in Children With Advanced Cancer: Assessment of the PediQUEST Study. *Journal of Pain and Symptom Management*, *49*(6), 1059–1069. 10.1016/j.jpainsymman.2014.12.010. 10.1016/j.jpainsymman.2014.12.010 PMC4530789 25640275

[37] Leahy, A. B., Feudtner, C., & Basch, E. (2018). Symptom monitoring in pediatric oncology using Patient-Reported outcomes: Why, how, and where next. *The Patient - Patient-Centered Outcomes Research*, *11*(2), 147–153. 10.1007/s40271-017-0279-z 29071524 10.1007/s40271-017-0279-z PMC5845473

[38] Goh, X. T. W., Tan, Y. B., Thirumoorthy, T., & Kwan, Y. H. (2017). A systematic review of factors that influence treatment adherence in paediatric oncology patients. *Journal of Clinical Pharmacy and Therapeutics*, *42*(1), 1–7. 10.1111/jcpt.12441 28045208 10.1111/jcpt.12441

[39] Morris, A. C., Macdonald, A., Moghraby, O., Stringaris, A., Hayes, R. D., Simonoff, E., & Downs, J. M. (2021). Sociodemographic factors associated with routine outcome monitoring: A historical cohort study of 28,382 young people accessing child and adolescent mental health services. *Child and Adolescent Mental Health*, *26*(1), 56–64. 10.1111/camh.12396 32544982 10.1111/camh.12396

[40] Bhatia, S., Landier, W., Shangguan, M., Hageman, L., Schaible, A. N., Carter, A.R.,… Wong, F. L. (2012). Nonadherence to Oral Mercaptopurine and Risk of Relapse in Hispanic and Non-Hispanic White Children With Acute Lymphoblastic Leukemia: A Report From the Children’s Oncology Group. *Journal of Clinical Oncology*, *30*(17), 2094–2101. 10.1200/JCO.2011.38.9924. 10.1200/JCO.2011.38.9924 PMC3601449 22564992

[41] Psihogios, A. M., Fellmeth, H., Schwartz, L. A., & Barakat, L. P. (2019). Family functioning and medical adherence across children and adolescents with chronic health conditions: A Meta-Analysis. *Journal of Pediatric Psychology*, *44*(1), 84–97. 10.1093/jpepsy/jsy044 29982694 10.1093/jpepsy/jsy044 PMC7967873

[42] Bernstein, D. N., Karhade, A. V., Bono, C. M., Schwab, J. H., Harris, M. B., & Tobert, D. G. (2022). Sociodemographic factors are associated with Patient-Reported outcome measure completion in orthopaedic surgery: An analysis of completion rates and determinants. *JBJS Open Access*, *7*(3). 10.2106/JBJS.OA.22.00026 10.2106/JBJS.OA.22.00026 PMC9355105 35935603

[43] McCabe, E., Rabi, S., Bele, S., Zwicker, J. D., & Santana, M. J. (2023). Factors affecting implementation of patient-reported outcome and experience measures in a pediatric health system. *Journal of Patient-Reported Outcomes*, *7*(1), 24. 10.1186/s41687-023-00563-1 36892738 10.1186/s41687-023-00563-1 PMC9998780

[44] Sisodia, R. C., Dankers, C., Orav, J., Joseph, B., Meyers, P., Wright, P., & Sequist, T. D. (2020). Factors associated with increased collection of Patient-Reported outcomes within a large health care system. *JAMA Network Open*, *3*(4), e202764. 10.1001/jamanetworkopen.2020.2764 32286657 10.1001/jamanetworkopen.2020.2764 PMC7156989

[45] Mayrhuber, L., Andres, S. D., Legrand, M. L., Luft, A. R., Ryser, F., Gassert, R.,… Lambercy, O. (2024). Encouraging arm use in stroke survivors: the impact of smart reminders during a home-based intervention. *Journal of NeuroEngineering and Rehabilitation*, *21*(1), 220. 10.1186/s12984-024-01527-2. 10.1186/s12984-024-01527-2 PMC11662716 39707385

[46] Meryk, A., Kropshofer, G., Hetzer, B., Riedl, D., Lehmann, J., Rumpold, G., & Crazzolara, R. (2023). Disagreement between mothers’ and fathers’ rating of health-related quality of life in children with cancer. *Quality of Life Research*, *32*(6), 1683–1691. 10.1007/s11136-023-03341-0 36635414 10.1007/s11136-023-03341-0 PMC9836339

