# Supplementary material for: Adherence to daily electronic symptom screening in pediatric cancer: associations with time, location and pain
Source: Qual Life Res. 2025 Jun 10;34(8):2363–75. doi: 10.1007/s11136-025-04002-0 (PMC12274143; doi:10.1007/s11136-025-04002-0)
Supplement: Supplementary file 2 — Supplementary Material 2 [file 11136_2025_4002_MOESM2_ESM.docx]

# Supplementary Material 2

## Supplementary Material 2.1: Number of patients by CTCAE grade and total time with highest CTCAE grading per patient

|  | n | Percentage of patients that had this CTCAE grade | Mean number of days with this CTCAE grade | SD |
| --- | --- | --- | --- | --- |
| Any CTCAE Grade 1 | 50 | 100 | 20.94 | 13.83 |
| Any CTCAE Grade 2 | 49 | 98 | 41.28 | 27.42 |
| Any CTCAE Grade 3 | 43 | 86 | 23.82 | 23.68 |
| Any CTCAE Grade 4 | 15 | 30 | 1.90 | 4.95 |

## Supplementary Material 2.2: Days with CTCAE rating by type (total)

| **Days with CTCAE ratings by type** | Days not in the hospital (no rating conducted) | Grade 0 | Grade 1 | Grade 2 | Grade 3 | Grade 4 |
| --- | --- | --- | --- | --- | --- | --- |
| Clinically acute |  |  |  |  |  |  |
| Febrile neutropenia | 7031 | 4831 | NA^a^ | NA^a^ | 236 | 56 |
| Mucositis | 7031 | 4531 | 184 | 157 | 251 | 0 |
| Pneunomia | 7031 | 5056 | 17 | 10 | 5 | 35 |
| Laboratory diagnosed |  |  |  |  |  |  |
| Anaemia | 7031 | 550 | 1257 | 2479 | 837 | 0 |
| Hepatopathy | 7031 | 2669 | 1820 | 364 | 257 | 13 |

Note. ^a^there are no grade 1 or 2 ratings for FN

## Supplementary Material 2.3: Full Model

| Covariates | Multivariable Model (all covariates included) | | |
| --- | --- | --- | --- |
|  | β | [95% CI] | p-value |
| Constant of multivariable model | 0.5316 | [0.1749; 0.8883] | 0.004** |
| Age | -0.0065 | [-0.0201; 0.0071] | 0.357 |
| Sex | -0.0307 | [-0.1585; 0.0971] | 0.640 |
| Caregiver highest education | 0.0740 | [0.0018; 0.1462] | 0.052 |
| Urban residency | -0.0115 | [-0.1364; 0.1134] | 0.858 |
| Tyrol residency | 0.0532 | [-0.0623; 0.1686] | 0.374 |
| Single parent | 0.0467 | [-0.0796; 0.1730] | 0.474 |
| Number of siblings | -0.0295 | [-0.0888; 0.0299] | 0.337 |
| **Time in the program** | **-0.0672** | **[-0.1028; 0.0317]** | **0.0002**** |
| *Location of care* |  |  |  |
| At home | 0.0461 | [-0.4162; 0.5084] | 0.845 |
| Inpatient | 0.1469 | [-0.0184; 0.3122] | 0.083 |
| Unplanned hospitalization | 0.0722 | [-0.0972; 0.2415] | 0.405 |
| **PICU** | **-0.2372** | **[-0.3756; -0.0987]** | **0.0009**** |
| Ambulatory care | 0.1017 | [-0.0364; 0.2398] | 0.151 |
| CTCAE Events |  |  |  |
| Clinically acute CTCAE grade <=2 | 0.1128 | [-0.0320; 0.2576] | 0.129 |
| Clinically acute CTCAE grade >=3 | 0.0559 | [-0.0296; 0.1414] | 0.202 |
| Laboratory diagnosed CTCAE  grade <=2 | -0.0074 | [-0.1814; 0.1666] | 0.934 |
| Laboratory diagnosed CTCAE  grade >=3 | -0.0102 | [-0.0884; 0.0681] | 0.799 |
| *PROM scores* |  |  |  |
| PRO pain | 0.0927 | [-0.0251; 0.2105] | 0.125 |
| PRO nausea | 0.0520 | [-0.0596; 0.1636] | 0.362 |
| PRO sleep | 0.1073 | [-0.0752; 0.2897] | 0.251 |
| PRO physical functioning | 0.0028 | [-0.1018; 0.1073] | 0.959 |

## Note. Fit indices for the multivariable model Akaike Information Criterion = 32.8; Bayesian Information Criterion = 93.6; Log-Likelihood = 2.6; Marginal R² = 0.26; Conditional R² = 0.37 PRO = Patient Reported Outcomes; PROM = Patient Reported Outcome Measure; CTCAE = Common Terminology Criteria for Adverse Events; β = unstandardized regression coefficient; Significant variables are highlighted in bold.

## Supplementary Material 2.4: Adherence rate by age group

| **Age group (in years)** | N (50) | Mean adherence in % | SD |
| --- | --- | --- | --- |
| Daily PROM adherence^a^ |  | 48.7 | 27.2 |
| 5-8 | 17 | 50.3 | 31.0 |
| 8-12 | 14 | 52.6 | 30.8 |
| 12-18 | 19 | 44.4 | 20.9 |
| Weekly PROM adherence^b^ |  | 59.5 | 31.5 |
| 5-8 | 17 | 59.9 | 34.7 |
| 8-12 | 14 | 61.7 | 34.9 |
| 12-18 | 19 | 57.5 | 27.2 |

**Note.** PICU = Pediatric intensive care unit; ^a^In this analysis, adherence was considered on a daily basis per patient; ^b^In this sensitivity analysis, we considered adherence on a weekly basis with weeks with 3 assessments or more as adherent
